# Midline cervical bronchogenic cyst: A case report and literature review

**DOI:** 10.1097/MD.0000000000045417

**Published:** 2025-10-24

**Authors:** Yankun Chen, Yanling Shen, Ranran Li, Xiaojun Xue, Heqiang Qi, Song Zhou

**Affiliations:** a Department of General Surgery, The 909th Hospital, School of Medicine, Xiamen University. Zhangzhou, China; b Department of Cardiovascular Medicine, The 909th Hospital, School of Medicine, Xiamen University. Zhangzhou, China.

**Keywords:** BCs, ectopic, IONM, MRI, surgery

## Abstract

**Rationale:**

Bronchogenic cysts (BCs) are congenital anomalies typically located in the mediastinum or lung, with ectopic presentations in the neck being exceptionally rare. The potential for compressive symptoms and malignant transformation underscores the need for precise surgical resection.

**Patient concerns:**

A 53-year-old female presented with a self-palpable cervical mass and notable dysphagia. Medical imaging revealed a well-defined cystic lesion in the anterior cervical region.

**Diagnoses:**

The histopathological analysis post-resection confirmed a bronchogenic cyst lined by ciliated columnar epithelium consistent with ectopic foregut development.

**Interventions:**

Intraoperative neuromonitoring (IONM) was used to assist the complete resection of the cyst through the transcervical approach.

**Outcomes:**

The cyst was completely excised with preservation of vocal cord function intact. Postoperative follow-up over 12 months revealed no symptomatic recurrence, and imaging studies demonstrated no evidence of residual or recurrent lesions.

**Lessons:**

This clinical case highlights the criticality of considering BCs in cervical midline masses and demonstrates the efficacy of Magnetic resonance imaging (MRI) in differentiating ectopic BCs from adjacent tissues. Complete surgical resection combined with IONM optimized surgical safety, particularly in anatomically challenging regions near the recurrent laryngeal nerve (RLN).

## 1. Introduction

Bronchogenic cysts (BCs), a rare congenital anomaly arising from aberrant development of the foregut endoderm, have an incidence rate of 1/ 42 to 68,000, with some studies reporting even lower prevalence. Current evidence indicates a strong male predominance, while females account for around 20% of affected individuals.^[1]^ Based on their anatomical locations, BCs are broadly categorized into 3 types: intralobar, mediastinal, and ectopic. Over 99% of cases occur in the mediastinum or lung parenchyma, while ectopic subtypes including cervical ectopic cases account for <1% of total occurrences.^[2]^ BCs typically undergo progressive enlargement during growth phases, which increases the risk of compression, infection, and malignant transformation. These lesions are predominantly diagnosed in childhood, while adult-onset cases are relatively rare.^[3,4]^ To date, the pathogenesis of BCs remains poorly understood. Conventional physical examinations and imaging modalities only identify cystic lesions, hampering definitive diagnosis. In adults, cervical BCs may involve any cervical region, complicated by the complex anatomical topology of the neck and the inherent structural fragility of cyst walls. The synergy of these factors results in 2 important intraoperative risks. Firstly, intraoperative cyst wall rupture is associated with a heightened risk of postoperative recurrence. Secondly, accidental injury to the recurrent laryngeal nerve (RLN) may precipitate critical complications such as dyspnea and vocal cord dysfunction. Such complications significantly compromise patient quality of life.^[5]^ Through analysis of this case report, our research team aims to re-emphasize the prominence of diagnostic vigilance and therapeutic strategies for cervical ectopic BCs.

## 2. Case report

**Figure 1.**
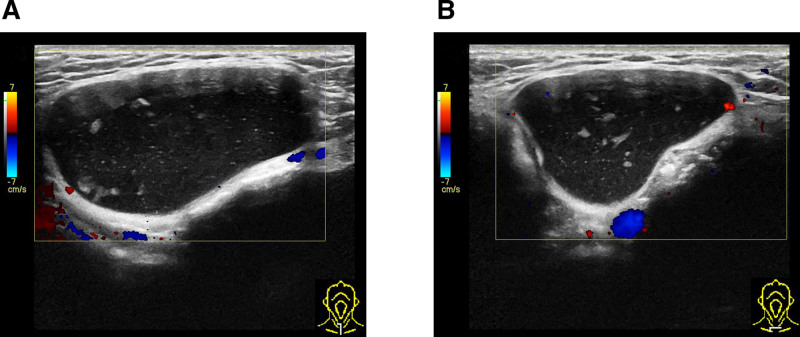
Multiangle ultrasonography revealed a well-circumscribed anechoic cervical mass containing hyperechoic debris. The ultrasound examination was performed using transverse and longitudinal scans in 2 orthogonal directions including craniocaudally (A) and radially (B).

**Figure 2.**
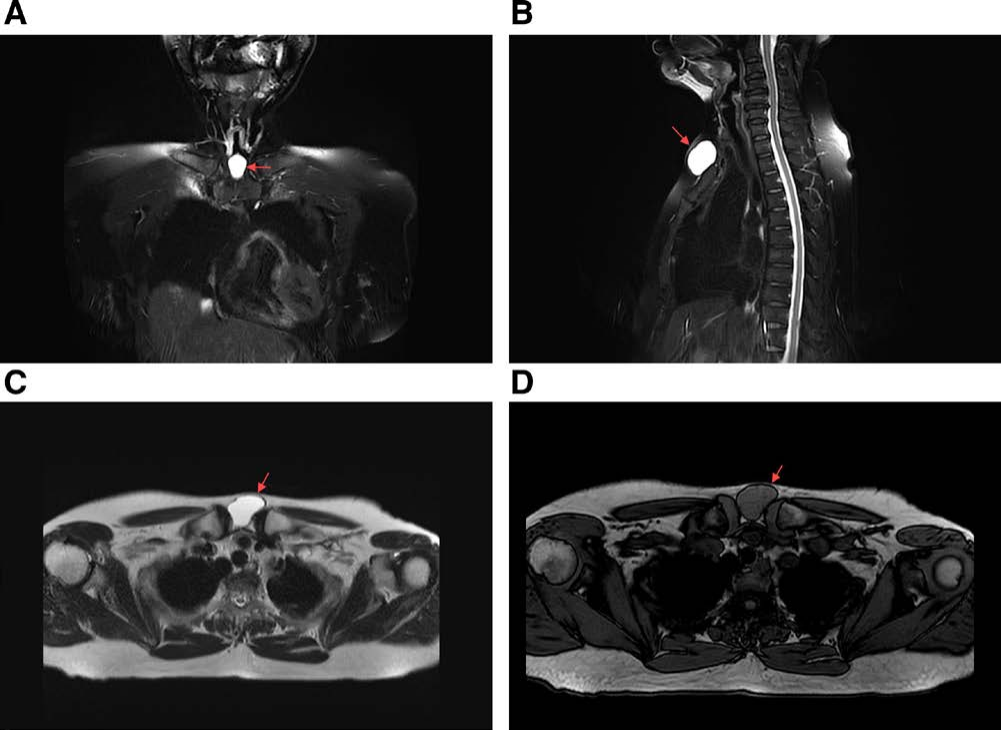
The cyst’s imaging anatomy was clearly identified through neck anteroposterior (A) and lateral (B) MRI examinations. Multiplanar magnetic resonance imaging findings demonstrated a cystic lesion exhibiting hypointense signals on T2-weighted imaging (C) and marked hyperintensity on T1-weighted sequences (D). The cystic mass was consistently demarcated by red arrows across all imaging series.

**Figure 3.**
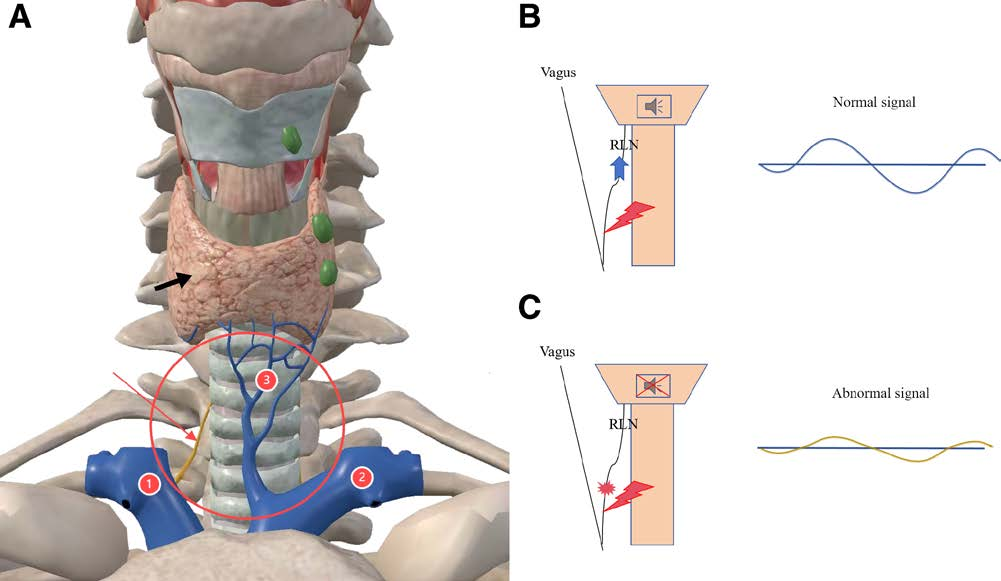
The red circular annotation demarcated the approximate cyst location, with red arrows indicating the right recurrent laryngeal nerve and black arrows delineating thyroid positioning. Labels 1 and 2 corresponded to the right and left brachiocephalic veins, respectively, while label 3 designated the inferior thyroid vein. (A) The IONM showed intact recurrent laryngeal nerves produced normal waveforms/signals when stimulated (B), while damaged nerves displayed abnormal responses (C). IONM = intraoperative neuromonitoring.

**Figure 4.**
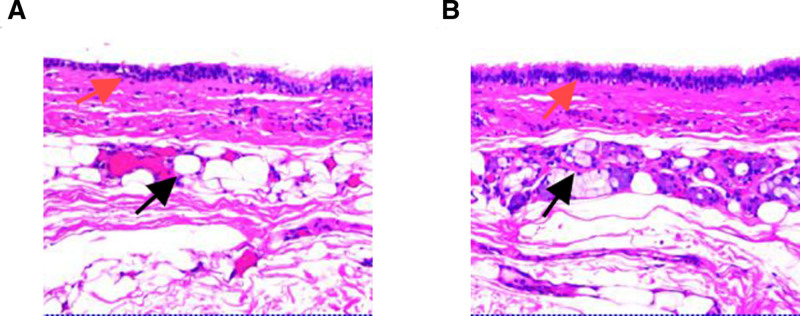
Histopathological assessment of the surgical specimen confirmed the coexistence of pseudostratified ciliated columnar epithelium (red arrows) and seromucinous glandular structures (black arrow) (A and B).

A 53-year-old female presented to our clinic on March 6, 2024, reporting a history of a cervical mass that had been present for at least 2 decades. She stated that the lesion was initially asymptomatic and undetectable during early adulthood, which explains her lack of medical consultation until the current admission. However, with progressive aging, the cervical mass demonstrated gradual enlargement. The patient has been experiencing a persistent foreign body sensation of recent onset, causing significant psychological distress. Persistent symptomatology coupled with visible cervical bulging ultimately prompted her family to seek specialized medical care at our institution. Physical examination revealed a localized protrusion in the anterior cervical region, with a palpable mass measuring 4 cm × 3 cm × 4 cm. The lesion demonstrated with smooth contours, well-defined borders, and moderately firm consistency, exhibiting moderate mobility. Palpation elicited no significant tenderness and fluctuation. The overlying skin showed no erythema or ulceration. On March 3, 2020, the patient underwent surgical resection of multiple colorectal polyps at our institution. Histopathological analysis confirmed a tubulovillous adenoma in the sigmoid colon with low-grade dysplasia. The patient persistently denied any significant personal medical history or familial predisposition to inherited disorders upon structured genealogical inquiry. Initial cervical ultrasonography identified a well-circumscribed, anechoic lesion in the suprasternal fossa, radiologically consistent with a subcutaneous cystic structure (Fig. 1A and B). To confirm diagnosis and facilitate surgical planning, magnetic resonance imaging (MRI) was performed on March 12, 2024. The mass demonstrated hypointensity on T1-weighted sequences and marked hyperintensity on T2-weighted sequences with fluid signal suppression, pathognomonic of a benign cystic lesion (Fig. 2A–D). Imaging characteristics strongly suggested a dermoid cyst. Preoperative laboratory evaluations including complete blood count, hepatic/renal function panels, and tumor markers (CEA, CA19-9, CA125) demonstrated values within reference ranges. The surgical team conducted systematic review of cervical fascial compartments using Netter anatomical schematics and implemented intraoperative neuromonitoring (NIM-Response® 3.0) (Fig. 3A–C). Under general anesthesia on March 13, 2024, complete en bloc resection of the neck mass was achieved, intraoperatively identifying a well-encapsulated cystic structure. Stepwise dissection using monopolar electrocautery (Valleylab™ FX8, 30W coagulation mode) was performed to circumferentially isolate the mass. IONM successfully mapped and preserved the right RLN, while no discernible neural structures were identified on the contralateral aspect. Because the cyst wall was relatively weak, the periphery was circumferentially packed with sterile gauze to prevent the cyst fluid extravasation and contamination of the surgical area. Regrettably, during the final stages of circumferential dissection, cyst rupture occurred with extravasation of opalescent mucoid material. Immediately used negative pressure suction to extract almost entirely cystic fluid. En bloc resection was then completed, with meticulous removal of all contaminated sterile gauze. Post-resection, pulsed lavage (1L normal saline alternating with 0.5% povidone-iodine) was administered, followed by precision fulguration of potential residual epithelial remnants using electrocautery. Histopathological evaluation demonstrated ciliated pseudostratified columnar epithelium lining the cyst wall, confirming the bronchogenic cyst diagnosis (Fig. 4A and B). Financial constraints precluded confirmatory immunohistochemical analysis. It is puzzling that after the operation, the patient had occasional choking-induced cough on drinking water, but there was no hoarseness, aphonia, dyspnea, or other symptoms. Mecobalamin was administered for 1 week, and the discomfort did not recur during the treatment and after discontinuation. The patient’s symptoms were significantly relieved postoperatively without specific adverse effects. No recurrence was observed during a 1-year follow-up, and the patient expressed satisfaction with the treatment outcome.

## 3. Discussion

The embryogenic mechanisms underlying BCs remain incompletely understood. Three hypotheses had historically been proposed to explain their embryological origins and pathogenesis. However, insufficient evidence persisted across multiple research dimensions, which made it challenging to construct a cohesive chain of supporting evidence. Prevailing studies predominantly explored the embryonic detachment and displacement hypothesis. This theory posited that lung buds originating from the primitive foregut initially developed as cord-like structures during early embryogenesis. Through progressive maturation, these cords evolved into tubular configurations, ultimately formed the bronchial tree and alveoli. Should developmental anomalies occur, the primitive bronchial tissue at the distal end separated from proximal structures and migrated to ectopic sites like the cervical region. The arrested transition from cord-like to tubular architecture resulted in blind-ended ducts. Secretions trapped within these isolated lumina gradually ‌accumulated, leading to mucus-filled cervical cysts that might exhibit features characteristic of any foregut derivative.^[6,7]^ The final anatomical positioning of BCs correlated directly with the timing of this developmental aberration. When branching abnormalities occurred prior to sternal membrane fusion, detached branches might develop into cysts anchored to thoracic structures. Conversely, disruptions arose post-sternal fusion typically led to cysts confined to subcutaneous tissue, with occasional migration into cephalad regions.^[8]^ By the time of this analysis, over 70 cases of BCs occurring in the head and neck region had been documented in the literature. Among cervical BCs, approximately 3-quarters localized to the midline cervical region. The remaining quarter predominantly occupied lateral cervical areas.^[9,10]^ This report detailed an adult female patient who presented with a midline neck mass in the lower cervical region, aligned with the previously described pattern. Notably, most adult bronchogenic cyst cases remained clinically asymptomatic, with lesions typically incidentally discovered during evaluations for unrelated conditions. Contrary to this norm, the current case involved a patient who proactively sought care for the palpable mass itself and reported noticeable discomfort exacerbated during swallowing – a deviation from the expected clinical trajectory. Even when a neck mass was clearly identified as a cystic lesion, it still posed significant challenges throughout the diagnostic and management process. Initially, the differential diagnosis posed significant complexity due to the extensive variety of cervical cystic lesions. A retrospective review of 331 neck cyst cases demonstrated that BCs accounted for <1% of presentations. Given this statistical context, our research team found it challenging to confirm this rare diagnosis and instead gravitated toward more prevalent etiologies such as branchial cleft cysts and dermoid cysts. At 1 point, there was transient suspicion that malignant transformation of a sigmoid colon villous tubular adenoma had triggered metastatic lymph node involvement in the neck.^[11,12]^ Moreover, none of the cystic lesions exhibited distinct imaging characteristics, and their radiographic presentations demonstrated significant overlap. This rendered our initial approach of relying solely on imaging diagnostics ineffective.^[13]^ Studies indicated that thyroid and paratracheal cysts were more likely to develop secondary infections through fibrous tract communications with the tracheobronchial system. This phenomenon frequently eluded detection on preoperative MRI and escaped identification during intraoperative assessment.^[14,15]^ The patient’s mass was located in the peri-thyroidal and paratracheal region. Failure to identify and ligate such fibrous connections intraoperatively could lead to protracted postoperative complications such as persistent infection. In such scenarios, revision surgery might become necessary, imposing additional physical and financial burdens on the patient.^[16]^ Percutaneous cyst aspiration with drainage had been proposed historically as an alternative to surgical excision. However, this approach never gained traction within clinical practice due to its documented high recurrence rates and a malignancy complication rate reaching 0.7%.^[17,18]^ Studies demonstrated marked variations in diagnostic approaches for BCs across different anatomical locations. MRI, however, remained the preferred imaging modality for confirming these lesions due to its superior soft tissue characterization.^[19,20]^ Given these clinical considerations, we recommended complete surgical excision as the definitive approach. This strategy achieved dual objectives: obtaining pathological confirmation for malignancy surveillance while allowing intraoperative assessment of potential cyst-airway communications. Preoperative MRI scans remained critical for delineating cyst dimensions and their anatomic relationships to adjacent structures, which directly informed operative planning.^[21]^ The cervical anatomy posed inherent surgical risks given the proximity of vital structures like the trachea and neural networks. Published reports documented RLN fibers maybe directly adherent to cyst walls in documented cases, necessitating operator expertise in regional anatomy to mitigate procedural complications.^[13,22]^ Intraoperative management protocols advocated meticulous cyst wall ablation via electrocautery when dense adhesions to adjacent tissues were encountered, prioritizing complete resection to minimize recurrence risks. This approach, however, carried inherent thermal injury potential to the RLN, as evidenced by postoperative aspiration events in this case attributed to intraoperative thermal trauma. To address this risk, meticulous RLN dissection combined with IONM was recommended to visually confirm nerve pathways and minimize thermal spread during cauterization.^[22,23]^ BCs were defined as developmental lesions histologically characterized by a respiratory epithelial lining that demonstrated architectural and cytologic features broadly identical to those observed in normal tracheobronchial mucosa. Past studies indicated that in all rare-location cysts except spinal BCs, the cystic linings showed positive staining for CK7, TTF-1, and p40, but were negative for CK20. However, due to limited sample sizes and few reports on ectopic cases, diagnostic blind spots persisted. Therefore, histopathological examination – with its irreplaceable morphological analysis – continued to be regarded as the gold standard for diagnosing BCs. Key features included a pseudostratified, ciliated, columnar epithelial lining with underlying seromucous glands. Which aligned perfectly with the postoperative pathology findings in this patient. Occasionally, cartilage or smooth muscle tissue might also be present.^[24,25]^ The clinical course of this disorder was characterized by a favorable prognosis and a low propensity for recurrence. In cases where relapse occurred, comprehensive assessment via physical examination coupled with advanced imaging modalities enabled definitive identification. Reassuringly, during the 1-year postoperative follow-up, the patient returned for evaluations every 6 months. No signs of recurrence were detected, and the patient recovered well.

## 4. Conclusion

Cervical BCs remained exceptionally uncommon clinical entities. When present, these lesions were typically located within the anterior cervical midline region. This anatomical predilection necessitated their inclusion in the differential diagnosis when encountering cystic masses in the mid-cervical area. Histopathological verification stood as the sole definitive diagnostic modality under such circumstances. The surgical management of these cysts carried significant technical challenges due to their frequent proximity to critical neurovascular structures. Complete surgical excision with adjunctive intraoperative neuromonitoring techniques emerged as the optimal therapeutic strategy. This approach achieved 3 principal objectives: confirmation of pathological diagnosis, symptomatic relief through mass reduction, and mitigation of potential malignant transformation risks.

## Author contributions

**Conceptualization:** Yankun Chen, Yanling Shen, Song Zhou.

**Supervision:** Yankun Chen, Xiaojun Xue, Heqiang Qi.

**Visualization:** Yankun Chen, Xiaojun Xue, Heqiang Qi.

**Writing – original draft:** Yankun Chen, Yanling Shen, Ranran Li, Song Zhou.

**Writing – review & editing:** Yankun Chen, Yanling Shen, Ranran Li, Song Zhou.
